# Factors associated with prolonged post-operative acute care length of stay in limb amputation patients in Saskatchewan, Canada

**DOI:** 10.1186/s12913-021-07163-z

**Published:** 2021-10-20

**Authors:** Samuel Kwaku Essien, Audrey Zucker-Levin

**Affiliations:** School of Rehabilitation Science, University of Saskatchewan, 104 Clinic Place, Saskatoon, SK S7N 2Z4 Canada

## Abstract

**Background:**

The effect of predisposing factors on post-operative acute care length of stay (POALOS) after lower extremity amputation (LEA) has been sparsely studied with reports largely focused on major (through/proximal to the ankle) LEA specifically due to diabetes mellitus (DM). Although valuable, the narrow focus disregards the impact of other causes and minor levels (distal to the ankle) of LEA. To address this gap, this study aimed to identify predisposing factors associated with prolonged POALOS after index LEA stratified by amputation level in Saskatchewan.

**Methods:**

The study used Saskatchewan’s provincial linked administrative health data and demographic factors between 2006 and 2019. Amputation levels, identified as major or minor, were derived from the amputation procedure codes. POALOS was calculated by subtracting patients’ intervention date from discharge date, recorded in days, and categorized as short (< 7 days) or prolonged (> 7 days). Multivariable logistic regression was performed to identify predictors associated with prolonged POALOS.

**Results:**

Of the 3123 LEA cases 1421 (45.5%) had prolonged POALOS. The median POALOS for the entire cohort was 7 days (IQR 3 to 16 days); 5 days (IQR 1 to 10 days) for minor LEA and 11 days (IQR 5 to 23 days) for major LEA. Predictors of prolonged POALOS after minor LEA were diabetes (AOR = 2.47, 95% CI: 1.87–3.27) and general surgeon (AOR = 1.52, 95% CI: 1.21–1.91). Minor LEA performed by orthopedic surgeons were half (AOR = 0.49, 95% CI: 0.35–0.70) as likely to experience prolonged POALOS. Predictors of prolonged POALOS after major LEA were diabetes (AOR = 1.34, 95% CI: 1.04–1.71), general surgeon (AOR = 1.91, 95% CI: 1.45–2.49), urban residence (AOR = 1.58, 95% CI: 1.25–1.99), Resident Indian (RI) status (AOR = 1.57, 95% CI: 1.15–2.15), and age with the likelihood of prolonged POALOS after LEA attenuating with increasing age: 35–54 years (AOR = 2.73, 95% CI: 1.56–4.76); 55–69 years (AOR = 2.65, 95% CI: 1.54–4.58); and 70+ years (AOR = 1.81, 95% CI: 1.05–3.11).

**Conclusion:**

This study identified only diabetes and surgical specialty predicted prolonged POALOS after both major and minor LEA in Saskatchewan while residence, RI status, and age were predictors of POALOS after major LEA. These findings shed light on the need for further research to identify confounding factors. It is not clear if general surgeons care for more unplanned, emergent cases with poor entry-level health while specialty surgeons perform more scheduled procedures.

## Introduction

Healthcare systems are evolving from a consumption-based model to a value-based model where patient outcomes and associated costs are scrutinized [1]. Decreasing acute hospital length of stay (LOS) assists with cost savings and may improve patient outcomes by decreasing hospital adverse events [2, 3]. In general, extended length LOS has been associated with poor entry-level patient health, co-morbidity, advanced age, inadequate social support, surgical specialty, socioeconomic status, type of hospital, type/level of amputation, and payment method [4–9].

The effect of predisposing factors on LOS, specifically after lower extremity amputation (LEA) has been sparsely studied with reports largely focused on LEA caused by diabetes mellitus (DM), the leading cause of LEA, and further amalgamating levels of amputation (major/minor) into a single cohort [10, 11]. For example, Franklin reported the overall median length of stay in 2010 was 11 days after lower-extremity amputation (LEA) in United States veterans with diabetes; median LOS for toe amputation was 8 days, 12 days for trans-tibial amputation, and 15 days for trans-femoral amputation [12]. Analysis of contributing factors to LOS was not described as the study focused on costs of care to the veteran’s health administration [12].

Among Canadian provinces, Saskatchewan has one of the highest average age-adjusted LEA rates (28.3 per 100,000) [13] and the second-highest per-capita indigenous population among Canadian provinces with 16.3% of the population self-identified as indigenous [14]. Our previous research identified that First Nations persons registered under the Indian Act of Canada experienced higher rates of limb amputation than the general population [15]. Also, 34% of the Saskatchewan population resides in rural areas, which is nearly double the 18.9% Canadian national average [16]. Our unique demographics facilitate studying the impact of these factors on POALOS after LEA. Presently there is only one published study that broadly examined the length of stay (LOS) after LEA in Canada from 2006 and 2009 [5]. This study specifically compares indications and discharge disposition after LEA among the Canadian provinces but does not include LEA due to trauma nor does it explore factors such as residence type or First Nation status that may influence LOS after LEA. This study aimed to identify predisposing factors associated with prolonged POALOS after LEA stratified by LEA levels (major/minor) in Saskatchewan from 2006 to 2019.

## Methods

### Data

The study used provincial retrospective administrative health data, accessed through the Saskatchewan Health Quality Council. The data consisted of hospital discharge records for index (first report of amputation in an individual) procedures via the use of the Canadian Classification of Health Interventions (CCI: 1SQ93, 1VA93, 1VC93, 1VG93, 1VQ93, 1WA93, 1WE93, 1WI93, 1WJ93, 1WK93, 1WL93, 1WM93, 1WN93) [17] for the period January 1, 2006, to December 31, 2019. The validity of the CCI codes was studied by Decoster et al. [18]. Although limb amputation was not examined among their procedures, the authors concluded that the CCI well-identified/coded major procedures but had a limitation in the validity of minor procedures such as lumbar puncture and gastric tube insertion [18]. CCI coding has been used for health care services and population health research, including to study LEA [15, 18, 19]. Further, these codes are updated regularly to accommodate changes to new intervention standards and practices [20].

In addition, information on the health facility type where surgery was performed, surgical specialty, amputation intervention date, discharge date was obtained from *hospital discharge abstract records (DAD),* and *Physician Characteristics and Mobility file*. The level of amputation, identified as major (through/above the ankle joint), or minor (below the ankle joint), was derived from the amputation procedure codes. The completeness, reliability, and validity of data are well documented [21, 22]. The University of Saskatchewan Biomedical Ethics Board (U of S #Bio 1590) approved the study.

### Outcome and explanatory variables

In this study, the outcome of interest investigated was POALOS after the first report of major or minor LEA. POALOS was calculated by subtracting patients’ intervention date from discharge date and recorded in days. To facilitate comparison, LOS was categorized as short (< 7 days) or prolonged (> 7 days) based on previous reports [5, 23].

For explanatory variable, patients’ demographic characteristics, including the location of residence, identified by postal code population (non-urban < 1000 and urban > 1000) [24, 25] and years 2018–2019 being a surrogate of the location of residence in 2017, age (0–34 years, 35–54 years, 55–69 years and 70+ years), sex (female/male) were retrieved from the *Person Health Registration System (PHRS)*. Age was grouped into four categories based on evidence in literature and knowledge in the field as LEA has been reported to increase after age 55 years [26]. Grouping age based on sample distribution is widely used in other fields [27], however applying this approach to amputation-related research may be misleading as some age groups (e.g., 0–10 years and 10–20 years) typically have a small number of samples. Our collective agreement with the data trustees prohibits reporting small numbers to ensure confidentiality.

Surgeon specialty included general surgeons, vascular surgeons, and orthopedic surgeons with small cell sizes limiting evaluation of LEA cases performed by other specialties. Type of health facility was categorized into provincial hospital (Regina general, Pasqua, St. Paul’s, Royal University, and City hospitals) and other health facilities (regional, district, community hospitals, and health care centers).

Diabetes was identified if diagnosed with chronic complications 5 years prior to the date of LEA using the Elixhauser comorbidity index [28] based on the International Classification of Disease (ICD) codes for diabetes complications including E10.2-E10.8, E11.2-E11.8, E12.2-E12.8, E13.2-E13.8, E14.2-E14.8 [29]. The diabetes complications index was then categorized as a binary indicator. Ethnicity cannot be identified in the Saskatchewan administrative databases, but it is possible to identify First Nations (FN) persons registered under the Indian Act of Canada [30], further referred to as Registered Indians (RI). Since 2010, Saskatchewan health care policies do not mandate individuals to declare their status but have the option to self-declare or not if they have FN status. The variable is not verified and reflects a self-declaration. People with RI status reported in the 2016 Canadian census was 106,440, a 60.8% of the 175,015 Indigenous residents of Saskatchewan [31]. All other Saskatchewan residents, including whites, immigrants, Indigenous peoples with registered Indian status who have not self-identified in the PHRS, non-registered Indian status, Metis and Inuits peoples, will be identified in the General Population (GP) cohort [15].

### Analysis

Demographic factors were described using proportions, and continuous variables were summarized as median and interquartile range. The study data was split into two broad levels of amputation (minor and major). This allowed for both POALOS and predictors to be compared between levels of amputation. A multiple logistic regression analysis [32, 33] was performed to ascertain the association between prolonged POALOS (> 7 days) and explanatory variables, with the overreaching goal of understanding the odds for an explanatory variable influencing prolonged POALOS after LEA. This method has successfully been applied and ascertained LOS in LEA cohorts in related studies published elsewhere [5, 23]. First, an unadjusted analysis was carried out for each explanatory variable, and all explanatory factors with *p*-values < 0.25 [32] qualified for inclusion in the adjusted model. Secondly, an adjusted model was fitted to all explanatory variables that met the inclusion criterion. The final adjusted model was achieved by eliminating all factors with *p*-values greater than the set significant level of 0.05 via the manual backward elimination method [32]. Potential confounders and interactions were assessed using guidelines stipulated in Hosmer et al.’s model-building strategies [32]. The odds ratio (OR) and the 95% confidence interval were reported for both the unadjusted and final adjusted models. The receiver operating characteristics (ROC) curve analysis was employed to compare models’ predictive power with different predictors [34].

## Results

Table 1 presents the demographic and outcome characteristics of all patients included in the current study. Of the 3123 LEA cases that met the study inclusion criteria, 1702 (54.5%) had a short POALOS, while 1421 (45.5%) had a prolonged POALOS. The median POALOS for the entire cohort was 7 days (IQR 3 to 16 days); 5 days (IQR 1 to 10 days), for the minor LEA cohort, and 11 days (IQR 5 to 23 days) for the major LEA cohort. Males and those older than 55 years constituted a larger proportion of patients in 2090 (66.9%) and 2309 (73.9%), respectively. Most (64.7%) patients had a diagnosis of diabetes 5 years prior to LEA, resided in an urban area (60.9%), and belong to the GP group (77.9%). Minor LEA cases were more common (56.3%) than major LEA cases (43.7%). Relatively, more cases were performed in the Provincial hospitals and by general surgeons (38.1%).

**Table 1:** Demographic and outcome characteristics of study population

Variables	Median	IQR	
LOS (days)		Lower	Upper
Entire Cohort	7	3	16
Major LEA Cohort	11	5	23
Minor LEA Cohort	5	1	10
**LOS Category (days)**	**N**	**%**	
Short (≤ 7)	1702	54.5	
Prolonged (> 7)	1421	45.5	
**Age (years)**			
0–34	211	6.8	
35–54	603	19.3	
55–69	1065	34.1	
70+	1244	39.8	
**Sex**			
Female	1033	33.1	
Male	2090	66.9	
**Diabetes**			
No	1103	35.3	
Yes	2020	64.7	
**Surgeon Specialty**			
Vascular	1059	33.9	
General	1191	38.1	
Orthopedic	873	28.0	
**Residence Types**			
Non-Urban	1222	39.1	
Urban	1901	60.9	
**Level of Amputation**			
Minor	1757	56.3	
Major	1366	43.7	
**Ethnicity**			
General Population (GP)	2434	77.9	
Registered Indian (RI)	689	22.1	
**Health Facility/ Hospital Type**			
Provincial	2720	87.1	
Other	403	12.9	

### IQR-interquartile range

The unadjusted model results of predictors of prolonged POALOS after LEA are described in Table 2. The results revealed that except for the sex of patients (*p* = 0.321) and the health facility/hospital (*p* = 0.063) in which the LEA was performed, the remainder of the explanatory variables were significantly associated with prolonged POALOS after LEA.

**Table 2:** Unadjusted model of predictors of prolonged POALOS after LEA

Variables	Unadjusted Odds Ratio (UOR)	95% CI	Overall ***P***-value
**Age (years)**			
0–34 (ref)			
35–54	2.69	(1.91–3.80)	
55–69	2.58	(1.86–3.57)	< 0.001
70+	2.09	(1.51–2.89)	
**Sex**			
Female (ref)			
Male	1.08	(0.93–1.25)	0.321
**Diabetes**			
No (ref)			
Yes	1.95	(1.68–2.27)	< 0.001
**Surgeon Specialty**			
Vascular (ref)			
General	1.44	(1.22–1.70)	< 0.001
Orthopedic	0.60	(0.50–0.73)	
**Residence Type**			
Rural (ref)			
Urban	1.25	(1.08–1.45)	0.003
**Level of Amputation**			
Minor (ref)			
Major	3.07	(2.65–3.56)	< 0.001
**Status**			
General Population (ref)			
Registered Indian (RI)	1.35	(1.14–1.60)	< 0.001
**Health Facility/ Hospital Type**			
Provincial (ref)			
Other	0.82	(0.66–1.01)	0.063

### CI-confidence interval

After adjusting for demographic factors and surgeon specialty, five factors, age, diabetes, surgeon specialty, residence type, and level of amputation, were found to be associated with prolonged POALOS after LEA (Table 3). Patients aged 35–54 years were 1.7 times (AOR = 1.7, 95% CI 1.17–2.45) more likely to have a POALOS after LEA compared to younger patients. Patients with a history of diabetes 5 years prior to their LEA operation were 1.9 times more likely to have POALOS after LEA than those without diabetes (AOR = 1.88, 95% CI 1.57–2.25). Likewise, patients whose LEA was performed by a general surgeon were more likely to have a POALOS after LEA than those performed by a vascular surgeon. However, the lower odds of having POALOS after LEA was found to be associated with LEA performed by an orthopedic surgeon (AOR = 0.72, 95% CI 0.58–0.90).

**Table 3:** Adjusted model of predictors of prolonged POALOS after LEA for the entire study cohort

Variables	Adjusted Odds Ratio (AOR)	95% CI	***P***-value
**Age/years**			
0–34 (ref)			
35–54	1.71	(1.17–2.48)	0.005
55–69	1.44	(0.99–2.07)	0.052
70+	1.05	(0.73–1.50)	0.809
**Diabetes**			
No (ref)			
Yes	1.88	(1.57–2.25)	< 0.001
**Surgeon Specialty**			
Vascular (ref)			
General	1.69	(1.41–2.01)	< 0.001
Orthopedic	0.72	(0.58–0.90)	0.003
**Residence Type**			
Non-Urban (ref)			
Urban	1.26	(1.08–1.48)	0.003
**Level of Amputation**			
Minor (ref)			
Major	3.82	(3.26–4.49)	< 0.001

Patients who resided in urban areas had 1.26 higher odds of POALOS after LEA than their counterparts in the non-urban area (AOR = 1.26, 95% CI 1.08–1.48). Finally, patients with major LEA were 3.8 times more likely to have POALOS than patients after minor LEA (AOR = 3.82, 95% CI 3.26–4.49).

Since evidence from Tables 1 and 3 shows that POALOS after LEA varies by level of amputation, all subsequent analyses focused on predictors’ differential impact on prolonged POALOS after LEA by the level of amputation. Table 4 shows a comparison between predictors of prolonged POALOS after major and minor LEA. Of the potential predictors adjusted for, only two factors, diabetes, and surgeon specialty predicted prolonged POALOS after minor LEA, whereas five factors: diabetes, surgeon specialty, age, residence type, and RI status, predicted prolonged POALOS after major LEA. In the minor LEA cohort, patients with diabetes were 2.47 times (AOR = 2.47, 95% CI 1.87–3.27) more likely to experience prolonged POALOS than non-diabetic patients and patients whose procedures were performed by general surgeons were 1.52 times (AOR = 1.52, 95% CI 1.21–1.91) more likely to experience prolonged POALOS. Patients whose minor LEA procedures were performed by orthopedic surgeons were 0.49 times less likely to experience prolonged POALOS when compared to vascular surgeons (AOR = 0.49, 95% Cl 0.35–0.70).

**Table 4:** Adjusted model of predictors of prolonged POALOS after LEA stratified by minor and major amputations

Variables	Adjusted Odds Ratio (AOR)	95% CI	***P***-value
**MINOR AMPUTATION COHORT**			
**Diabetes**			
No (ref)			
Yes	2.47	(1.87–3.27)	< 0.001
**Surgeon Specialty**			
Vascular (ref)			
General	1.52	(1.21–1.91)	< 0.001
Orthopedic	0.49	(0.35–0.70)	< 0.001
**MAJOR AMPUTATION COHORT**			
**Age/years**			
0–34 (ref)			
35–54	2.73	(1.56–4.76)	< 0.001
55–69	2.65	(1.54–4.58)	< 0.001
70+	1.81	(1.05–3.11)	0.033
**Diabetes**			
No (ref)			
Yes	1.34	(1.04–1.71)	0.021
**Surgeon Specialty**			
Vascular (ref)			
General	1.91	(1.45–2.49)	< 0.001
Orthopedic	1.03	(0.76–1.38)	0.870
**Residence Type**			
Non-Urban (ref)			
Urban	1.58	(1.25–1.99)	< 0.001
**Status**			
General Population (ref)			
Registered Indian (RI)	1.57	(1.15–2.15)	0.004

In the major LEA cohort, we found that POALOS was prolonged in all age groups compared to the 0–34-year-old group with AOR attenuating with increasing age. The magnitude of the odds of POALOS after major LEA was 1.8–2.7 times higher in patients 35 years and older compared to those 0–34 years (AOR = 2.73, 95% CI 1.56–4.76; AOR = 2.65, 95% CI 1.54–4.58; AOR = 1.81, 95% CI 1.05–3.11). Patients with diabetes were 1.34 times (AOR = 1.34, 95% CI 1.04–1.71) more likely to experience prolonged POALOS after major LEA than non-diabetic patients; procedures performed by general surgeons were 1.91 times (AOR = 1.91, 95% CI 1.45–2.49) more likely to result in prolonged POALOS after major LEA than those performed by vascular surgeons; urban residence were 1.58 times (AOR = 1.58, 95% CI 1.25–1.99) more likely to experience prolonged POALOS after major LEA than their non-urban counterparts and people with RI status were 1.57 times (AOR = 1.57, 95% CI 1.15–2.15) more likely to experience prolonged POALOS after major LEA when compared to the general population.

**Fig. 1:**
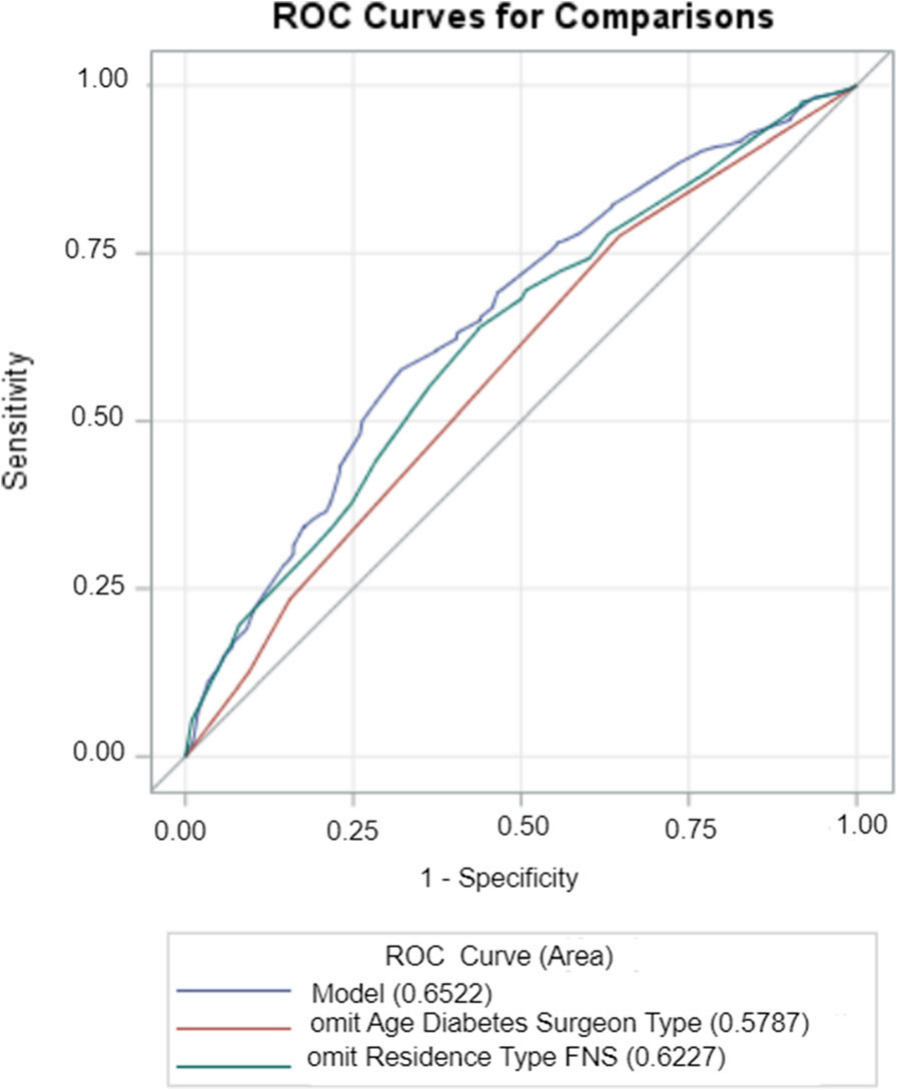
A plot comparing the predictive power of combined predictors of prolonged POALOS after major LEA

Receiver operating characteristics (ROC) curve analysis for predictors in the major LEA cohort (Fig. 1) revealed age, history of diabetes, and surgeon specialty were stronger predictors of prolonged POALOS after major LEA than residence type and RI status. This was evidence by their respective area under the curve values, AUC = 0.6227 and AUC = 0.5787, compared to the AUC = 0.6522 for the baseline model containing all five predictors.

## Discussion

We found a median POALOS of 7 days (IQR 3 to 16 days) after LEA for the period of 14 years (2006–2019); POALOS was 5 days (IQR 1 to 10 days) after minor LEA, and 11 days (IQR 5 to 23 days) after major LEA. When all LEA cases were considered, age, history of diabetes, surgeon specialty, residence type, and level of amputation were significantly associated with prolonged (> 7 days) POALOS while sex and type of health facility did not associate with prolonged POALOS. However, stratifying the study data by level of amputation (minor LEA and major LEA) revealed diabetes and specialty of surgeon predicted prolonged POALOS in both minor and major LEA with; age, residence type, and RI status also predictive of prolonged POALOS after major LEA. Moreover, the receiver operating characteristics (ROC) curve analysis for the major LEA cohort predictors revealed that a model with combined factors of age, history of diabetes, and surgeon specialty was a stronger predictor of prolonged POALOS after major LEA than the model with residence type and RI status as predictors.

The longer median POALOS after major LEA was not surprising and is consistent with other internationally published studies [8, 12, 35, 36]. Compared to findings from our study, a sample of US veterans with diabetes experienced slightly longer LOS after minor and major LEA [12]. Dillingham et al. found prolonged LOS for major LEA (about six times higher) than in patients with minor LEA [8]. Likewise, Ozan et al. found a significantly longer LOS after major LEA than minor LEA [35]. A Canadian report revealed that the average LOS after major LEA is quantified to be 10 times longer compared to minor LEA [36]. Although these findings are consistent with ours, an exact comparison cannot be made as we examined POALOS, while they examined total hospital LOS.

We were surprised to find higher odds of prolonged POALOS in patients residing in urban areas, specifically after major LEA. A recent study identified that specialized services, such as those for chronic disease management, including for diabetes, are often more difficult for rural Canadians to access [37]. Our cohort was made up of 39.1% non-urban residents and 64.7% of our cohort was diagnosed with diabetes. For this reason, we expected patients residing in non-urban areas would have more complicated medical needs necessitating prolonged POALOS after LEA. Possible explanations for this finding include the migration of typical non-urban residents in need of specialized care relocating to urban areas during the pre-operative period to access care closer to major medical facilities and long-term care homes, which would skew the true residence data. Also, our study focused on POALOS, not the entire hospital LOS. Patients in need of more medical attention because of limitations in specialized service delivery may have spent more time in the acute care hospital prior to the LEA procedure.

Unexpectedly, our adjusted model predicted prolonged POALOS in patients aged 35–54 years with age over 54 years, not a predictor of prolonged POALOS in the entire cohort. This non-significant association between patients aged 55+ years and prolonged POALOS in the entire cohort may in part be attributed to attenuation caused by the substantially larger proportion of minor LEA samples, in which age was not a predictor of prolonged POALOS. In contrast, age was a predictor of prolonged POALOS after major LEA. Those aged 35–54 were at the highest (2.73 times) risk followed by those aged 55–69 (2.65 times) and 70+ (1.81 times) for prolonged POALOS after major LEA. This diminishing risk with increasing age may be due to the increased severity of disease [38] when diabetes, the leading cause of LEA, is diagnosed between 18 and 44 years of age. These findings are similar to Kurichi et al., who found older age was a longer predictor of acute care LOS after major LEA [9].

We found 22.1% of LEA were performed in people with RI status and that they were 1.57 times more likely to have extended POALOS after major LEA but no difference in POALOS was identified after minor LEA. These findings are significant as Indigenous people account for 16.3% of the Saskatchewan population, with First Nations people accounting for 60.8% of Saskatchewan’s indigenous population [31]. This is of particular concern as Indigenous Canadians are at 4 times greater risk of getting diabetes [39].

Our finding that diabetes was associated with prolonged POALOS after LEA in all three adjusted models, specifically 1.9 times more likely after LEA in the entire study cohort: 2.47 times more likely in the minor and 1.34 times more likely in the major LEA cohort was not surprising and is consistent with other published studies [5, 36, 40, 41].

Surgical specialty influenced POALOS in our cohort. Patients whose procedures were performed by general surgeons were 1.91 times more likely (for major LEA) and 1.52 times more likely (for minor LEA) to have a prolonged POALOS compared to those performed by vascular surgeons. In contrast, when minor LEA was performed by an orthopedic surgeon patients were 0.49 times less likely to have prolonged POALOS. It is not clear if this observation is due to a difference in the cause of LEA. For example, general surgeons may care for more unplanned, emergent cases with poor entry-level health while specialty surgeons perform more scheduled procedures. Our observations are similar to those of Kayssi et al., who found people whose LEA was performed by a general surgeon were 1.5 times more likely to have prolonged post-operative LOS [5]. Kayssi et al. attributed this to the less familiar/experience general surgeon may have with the complex process of discharging postoperative LEA patients [5]. Interestingly, Kayssi found LOS after LEA was significantly lower (OR: 0.51, 95% CI: 0.38–0.70), when performed in Saskatchewan when compared to Ontario with no explanation or discussion of this finding.

### Strengths and limitations

This is the first study to identify specific factors that predict prolonged POALOS major and minor LEA in Saskatchewan. This study’s results are generalizable to all Canadian provinces/territories and other regions around the world especially those with similar demographic/geographical distribution as Saskatchewan. Due to the differential impact level of LEA may have on POALOS, it was a strength that the current study explicitly explored varying POALOS by the level of amputation. In addition, this study considered a broad range of predictors.

As for limitations, the non-urban and urban variable used in this study constitutes a limitation as misclassification of individuals who might have an urban mailing address but a non-urban residence; likewise, individuals might reside in temporary urban settings pre- amputation to receive intervention not available near their rural dwellings. Also, we recognize that the data available to identify the indigenous population only accounts for 60.8% of First Nations people in Saskatchewan. For this reason, our finding that people with RI status are 1.35 times more likely to experience prolonged POALOS after LEA, specifically major LEA is likely an underestimation. Although the quality of administrative health data based in Canada is ranked high [42], coding of diagnosis and intervention procedures may be impacted by several issues including non-specific diagnoses, incomplete charts, and diagnosis typing [43, 44]. Other comorbidities including hypertension, congestive heart failure, and ischemic heart disease found to be associated with both LEA and LOS [5] were not adjusted for in this study. Finally, there is the potential for selection bias due to the exclusion of LEA cases performed by other surgical specialties. However, this might have had a minimal or insignificant impact on the study’s findings as only limited samples were excluded.

## Conclusion

This study identifies specific factors that predict prolonged POALOS after major and minor LEA in Saskatchewan. We found that history of diabetes and surgeon specialty predicted prolonged POALOS for both major and minor LEA with age, residence type, and Registered Indian status, predicting prolonged POALOS after major LEA. These findings shed light on the need for further research to determine if predictive factors, such as the surgeon’s specialty, have confounding factors. For example, it is not clear if patients cared for by general surgeons are sicker with less pre-op intervention than those cared for by other types of surgeons.

## Data Availability

The datasets used and/or analyzed during the current study are available from the corresponding author on reasonable request.

## Abbreviations

CI: Confidence Interval
OR: Odds Ratio
POALOS: Post Operative Acute care Length of Stay
LOS: Length of Stay
DM: Diabetes Mellitus
LEA: Lower Extremity Amputation
DAD: Discharge Abstract Records
PHRS: Person Health Registration System
FN: First Nation
RI: Registered Indian
GP: General Population
ROC: Receiver Operating Characteristics
IQR: Interquartile Range
